# An analysis of omega-3 clinical trials and a call for personalized supplementation for dementia prevention

**DOI:** 10.1080/14737175.2024.2313547

**Published:** 2024-02-20

**Authors:** Nicolás Castellanos-Perilla, Miguel Germán Borda, Dag Aarsland, George E. Barreto

**Affiliations:** a Department of Clinical Medicine, University of Bergen, Bergen, Norway; b Centre for Age-Related Medicine (SESAM), Stavanger University Hospital, Stavanger, Norway; c Semillero de Neurociencias y Envejecimiento, Ageing Institute, Medical School, Pontificia Universidad Javeriana, Bogotá, Colombia; d Department of Neurobiology, Care Sciences and Society, Karolinska Institutet, Stockholm, Sweden; e Division of Clinical Geriatrics, Center for Alzheimer Research, Department of Neurobiology, Care Sciences and Society, Karolinska Institutet, Stockholm, Sweden; f Department of Old Age Psychiatry, Institute of Psychiatry, Psychology, and Neuroscience, King’s College London, London, UK; g Department of Biological Sciences, University of Limerick, Limerick, Ireland

**Keywords:** Neurodegenerative diseases, Alzheimer’s disease, dementia, fatty acids, omega-3, dietary supplements, precision medicine

## Abstract

**Introduction:**

Targeted interventions are needed to delay or prevent the onset of neurodegenerative diseases. Poor dietary habits are associated with cognitive decline, highlighting the benefits of a healthy diet with fish and polyunsaturated fatty acids (PUFAs). Intake of omega-3 PUFAs docosahexaenoic acid (DHA), α-linolenic acid (ALA) and eicosapentaenoic acid (EPA) is linked with healthy aging, cardiovascular benefits, and reduced risk of Alzheimer’s disease. Although omega-3 has health benefits, its intake is often inadequate and insufficient in modern diets. Although fish oil supplements offer an alternative source, inconsistent results from clinical trials raise questions about the factors determining their success.

**Areas covered:**

In this this review, the authors discuss the aforementioned determining factors and highlight strategies that could enhance the effectiveness of omega-3 PUFAs interventions for dementia and cognitive decline. Moreover, the authors provide suggestions for potential future research.

**Expert opinion:**

Factors such as diet, lifestyle, and genetic predisposition can all influence the effectiveness of omega-3 supplementation. When implementing clinical trials, it is crucial to consider these factors and recognize their potential impact on the interpretation of results. It is important to study each variable independently and the interactions between them.

## 1. Introduction

The prevalence of Alzheimer’s disease (AD) [1]. This suggests that current therapeutic interventions are not effective in mitigating the pathology. Therefore, there is an urgent need to implement more precise therapies. Although curative treatments remain non-existent and disease-modifying treatments have had limited success, some nutritional strategies have the potential to delay or even prevent the onset of the disease. There is a significant correlation between unhealthy dietary habits and cognitive decline associated with AD. For instance, inadequate intake of fish oil has been linked to negative health outcomes and an increased likelihood of cognitive impairment [2–4], suggesting that consuming fish oil and polyunsaturated fatty acids (PUFAs) may help to lower the risk of cognitive decline [2,4–6].

Omega-3 PUFAs exert health benefits and have been shown to lower disease risk [7]. These PUFAs have been linked to healthy aging, cardiovascular health, and a reduced risk of AD [8]. They also contribute to maintaining cell membrane integrity and possess anti-inflammatory properties [9,10]. Unfortunately, modern diets contain less omega-3 fatty acids (FAs) and a higher proportion of omega-6 PUFAs. As a result, excessive consumption of omega-6 from the diet may be involved in the underlying mechanisms of several illnesses such as cancer, inflammatory and autoimmune disorders, as well as cardiovascular diseases [11]. As the human body cannot synthesize omega-3 fatty acids, these must be obtained through diet [12,13], primarily from seafood sources such as fish [14,15]. Fish oil supplements provide similar blood fatty acid levels compared to fish consumption [16]. Docosahexaenoic acid (DHA), the predominant omega-3 PUFAs in the brain, is crucial in controlling the expression of inflammatory genes and amyloid-β (Aβ) in AD, highlighting the importance of obtaining it from circulating FA pools due to the brain’s limited capacity for synthesis [17].

Arachidonic acid (AA), a major omega-6 PUFAs, is converted into potent inflammatory lipid mediators such as thromboxanes, prostaglandins, and leukotrienes [18]. One of the pathways PUFAs are involved in inflammation is through the stimulator of interferon genes (STING), a key controller of nucleic acid-associated inflammatory responses, where PUFAs (omega-3 and omega-6) exert an inhibitory effect on STING-dependent inflammatory response [19]. Omega-3 PUFAs compete with omega-6 for incorporation into all cell membranes. This competition affects arachidonic acid metabolism by displacing it from the cellular membranes and competing with enzymes responsible for synthesizing thromboxanes, prostaglandins, and leukotrienes [10]. Thanks to the membrane incorporation of omega-3 PUFAs, the correlation between dietary intake and erythrocyte FA levels was stronger compared to plasma, especially DHA [20].

Omega-3 PUFAs are potent regulators of the immune system. The accumulation of toxic Aβ species leads to the formation of insoluble plaques, which activate the complement system and cause neuronal atrophy [21]. Notably, supplementation with omega-3 PUFAs has been shown to interact with the key components of the complement cascade, including complement 5 (C5), S100 Calcium Binding Protein A9 (S100A9), Complement C1q B Chain (C1QB), and coagulation Factor V (F5). For example, omega-3 and cognition are positively associated with F5 and C1QB, suggesting its potential role in modifying cognition through complement-mediated synaptic pruning [22]. In AD, the prolonged activation of microglia results in significant neuronal damage due to the accumulation of Aβ peptide. This accumulation overwhelms innate defenses because of the reduced phagocytic activity and Aβ clearance [21]. Omega-3, especially DHA, might modulate the immune response of microglia. This is suggested by alterations in microglial morphology in mouse models of AD fed with a safflower oil diet, as well as in wild-type mice fed a fish oil diet. Reduced microglial counts were evident in the CA1, CA3, and DG (dentate gyrus of the hippocampus) regions [23]. In addition, regulatory T cells (Tregs), a type of peripheral lymphocytes, play a crucial role in inducing microglia and astrocyte phagocytic activity. This induction may contribute to clearing amyloid-β deposits in the early stages of AD [21,24]. In this regard, omega-3 fatty acids, DHA, EPA, and ALA, have been found to inhibit the activation of immune cells and promote specific functions, such as phagocytosis and Treg differentiation [25], which may aid in the clearance of Aβ Furthermore, DHA has been shown to reduce inflammasome activation by promoting autophagy in macrophages [26].

Despite the extensive research on omega-3 supplementation, inconsistencies in clinical trials persist [27], questioning the determinants for the successful or failed use of this supplementation for dementia and cognitive decline. Sex-dependent factors including hormone levels, body composition, and brain penetration should be considered since they can cause notable variations in PUFA levels, which can affect the outcomes of PUFAs-targeting therapies [28]. Understanding these differences may provide further insights into the effectiveness of omega-3 interventions and help develop more targeted approaches for preventing cognitive decline [28]. This review aims to shed light on these potential factors that contribute to the contradictory evidence found in studies exploring the same substance. To understand the role of PUFA in dementia and cognitive decline, we discuss the challenges observed in clinical trials and suggest strategies to enhance the efficacy of these interventions. As a search strategy, we retrieved the literature on PubMed using the MeSH terms ‘fatty acids, omega 3’ AND (‘neurodegenerative diseases’, ‘Alzheimer Disease’, ‘Dietary Supplements’). The search was limited to the five years following the publication of the most recent review on this topic by Canhada et al. [27]. We specifically sought studies for which at least the abstract was available in English. Our search strategy retrieved a total of 10 papers published after the previous review. After applying the inclusion and exclusion criteria, six new studies were included in this review. The findings of these studies were examined and compared with earlier previous literature (Table 1).

**Table 1.** Summary of clinical trials.

Study	Methods	Participants Diagnosis Sample (initial/final) Sex Age (mean)	Intervention	Outcomes
Lin et al. [29].	24 months RCT	AD (clinical) 163/131, 33% (F) 77.8 (treatment) vs 78.1 (placebo)	700 mg of DHA and 1600 mg of EPA daily or 350 mg of DHA and 800 mg of EPA daily. Placebo	EPA was found to decrease the levels of macrophage inflammatory protein (CCL4). Furthermore, EPA enhanced the scores for spoken language ability, while DHA improved the spoken language component of the ADAS-cog assessment.
Arellanes et al. [30].	6 months RCT	MCI 33/26 (for lumbar punctures and MRIs), 33/29 (for cognitive assessments), 81% (F) 68.5 (treatment), 69 (placebo).	Vitamin B + 2,152 mg of DHA daily Vitamin B + placebo	A 28% increase in CSF DHA and a 43% increase in CSF EPA were observed in the treatment group compared to the placebo group. Non-APOE4 carriers exhibited a threefold increase in CSF levels. Brain volumes and cognitive scores showed no significant differences between treatment and placebo.
Torres-Mendoza et al. [31].	12 months RCT	AD (clinical) 87/17 Unknown Unknown	Fish oil (450 mg of EPA and 1000 mg of DHA) Placebo	At month 6 and 12, carbonyl groups in plasma proteins decreased significantly in the fish oil group. Catalase activity was significantly higher at 6 and 12 months in comparison with the placebo group.
Tamtaji et al. [32].	12 weeks RCT	PD (clinical) 50/44 Unkonwn 65.5 (treatment) vs 67.7 (placebo)	1000 mg daily of omega-3 fatty acids from flaxseed oil + 400 IU/day of vitamin E Placebo	TNF-alpha gene expression was decreased in peripheral blood mononuclear cells (PBMC) with a significance of *p* = 0.002. The intake of omega-3 fatty acids and vitamin E resulted in an increase of oxidized low-density lipoprotein receptor (LDLR) (*p* = 0.002) and a decrease of unoxidized low-density lipoprotein receptor (PPAR-) in PBMC when compared to the placebo group.
Freund-Levi et al. [33]	6 months RCT, double blind	Mild-moderate AD 204/174 (85%) 51% (F) 72.6 (treatment) vs 72.9 (placebo)	DHA 430 mg + EPA 150 mg, daily. Group isocaloric placebo oil ((1 g of corn oil, including 0.6 g of linoleic acid Both groups received 4 mg of vitamin E (tocopherol)	The very mild cognitive dysfunction group (MMSE >27 points) experienced a reduced decline rate (MMSE), ranging from 0.5 to 1 (−0.5 points in the active group compared to −2.6 points in the placebo group; *p* = 0.01).
Chiu et al. [34]	24 weeks RCT, double blind	Mild or moderate severity AD 46/29 (63.04%), 57% (F) 74 (treatment) vs 76.5 (placebo)	EPA 1080 mg + DHA 720 mg, daily. Placebo: olive oil esters (dosage not informed).	The ADAS-cog improved in MCI, but not in AD, with a statistical significance of *p* = 0.03 (−3.23 ± 3.82 vs. −0.37 ± 1.4). The relative improvement in the CIBIC-plus score in the treatment group was −0.35 (−0.61 to −0.09, *p* = 0.008) after 6 weeks.
Quinn et al. [35]	18 months RCT, double blind	402/295 (73.4%), 52.2% (F)	DHA 2000 mg daily Corn or soy oil	ADAS-cog, CDR, ADCS-ADL, NPI, or MMSE did not demonstrate a significant difference after 18 months, and there was no significant difference in brain atrophy.
Scheltens et al. [36]	24 weeks, double blind	259/238 (92%), 49% (F)	DHA at a dosage of 1200 mg and EPA at a dosage of 300 mg, along with phospholipids, choline, UMP, vitamin B12, B6, and folate, as well as vitamins C and E, and selenium from Souvenaid®. Isocaloric control product	At 24 weeks no statistically significant difference in cognitive and functional abilities.
Soininen [37]	36 months, double blind		Souvenaid, a 125 mL	Reduced rate of brain atrophy, reduction in decline in the Neuropsychological Test Battery (−60%; *p* = 0.014), and function. memory (−76%; *p* = 0.008), as well as markers of brain shrinkage; Cohen’s d effect size (0.25–0.31) was minor to medium.
Shah et al. [38]	24 weeks, double blind	527/451(85%), 52% (F)	DHA 1200 mg + EPA 300 mg + phospholipids, choline, UMP, vitamin B12, B6, and folate, vitamins C and E, and selenium from Souvenaid® Isocaloric control product	Did not exhibit a decelerating effect on the cognitive function decline among individuals undergoing treatment for mild to severe Alzheimer’s disease (AD). Souvenaid demonstrated good tolerance when administered alongside other conventional AD medications.
Shinto et al. [39]	12 months, double blind	39/34 (87%), 43% (F)	Fish oil concentrate DHA 675 mg, EPA 975 mg vs omega-3 fatty acids same dose (DHA+EPA) + lipoic acid (600 mg/day) Soy oil	A statistically significant distinction was observed in the rate of decline of MMSE over 12 months between the placebo group and the group receiving omega-3 and lipoic acid. Regarding IADL, there was a notable mean change.
Phillips et al. [40]		19/19 (100%), 55% (F)	DHA 625 mg DHA + 600 mg EPA+ 20 mg mixed tocopherols, daily	There were no statistically significant differences between groups regarding the results of the MMSES7 (*p* = 0.711), MMSEWB (*p* = 0.576), and BADLS (*p* = 0.595) after a period of 4 months.

Alzheimer’s disease (AD), Alzheimer Disease Assessment Scale-cognitive subscale (ADAS-cog), Alzheimer’s Disease Cooperative Study (ADCS), Activities of Daily Living/Instrumental Activities of Daily Living (ADL/IADL), Bristol’s Activities of Daily Living Scale (BADLS), Clinical Dementia Rating (CDR), Clinician’s Interview-Based Impression of Change scale including caregiver-supplied information (CIBIC-plus), Hamilton Depression Scale (HDRS), Mild Cognitive Impairment (MCI), Mini-Mental State Examination (MMSE), Mini-Mental State Examination Serial Sevens (MMSES7), Mini-Mental State Examination World Backwards (MMSEWB), Neuropsychiatric Inventory (NPI), Neuropsychological Test Battery (NTB), Parkinson’s disease (PD), Randomized Controlled Clinical Trial (RCT).

## 2. Omega 3-metabolism

**Figure 1.**
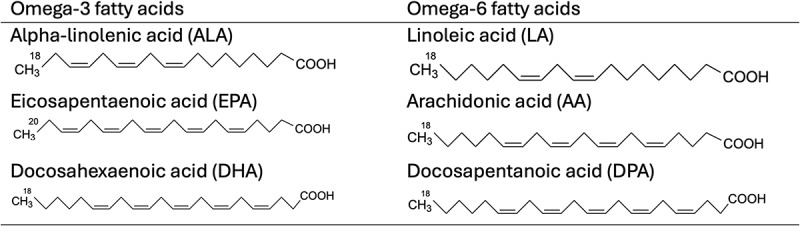
Omega-3 and omega-6 fatty acids chemical structure.

Long-chain omega-3 PUFAs eicosapentaenoic acid (EPA), docosapentaenoic acid (DPA), and DHA are derived from alpha-linolenic acid (ALA) through desaturation, elongation, and beta-oxidation processes (Figure 1). For proper digestion and absorption of fatty acids (FAs), the bile is secreted into the small bowel which emulsifies fat, ALA is then absorbed as triglycerides and then transported through plasma. This is followed by the esterification of phospholipids and triglycerides and oxidation (for ATP production) and forms a longer chain and saturated products. FAs are hydrolyzed by pancreatic carboxylic acid and ester lipase into free FAs, these are first absorbed in the small intestine, then bound to FA binding proteins, and finally incorporated into chylomicrons which are later secreted into lymph [41].

PUFAs concentrations in the body are influenced by various factors including their interaction with plasma proteins and their distribution in different cellular membranes. EPA concentrations are thought to be dependent on their interaction with plasma proteins, while DPA and DHA are more concentrated in the inner membrane and rely on erythrocyte turnover. A recent study provided evidence that fish oil supplementation increases EPA levels more than DHA, potentially due to differing rates of incorporation rates into membrane phospholipids. While EPA supplementation increases levels in plasma and erythrocytes, it does not affect plasma triacylglycerols [42]. These differences in incorporation rates are also observed in adipose tissue [43]. Variations in these rates of incorporation is important for understanding the complex interplay of PUFAs and their diverse effects on various body tissues.

**Figure 2.**
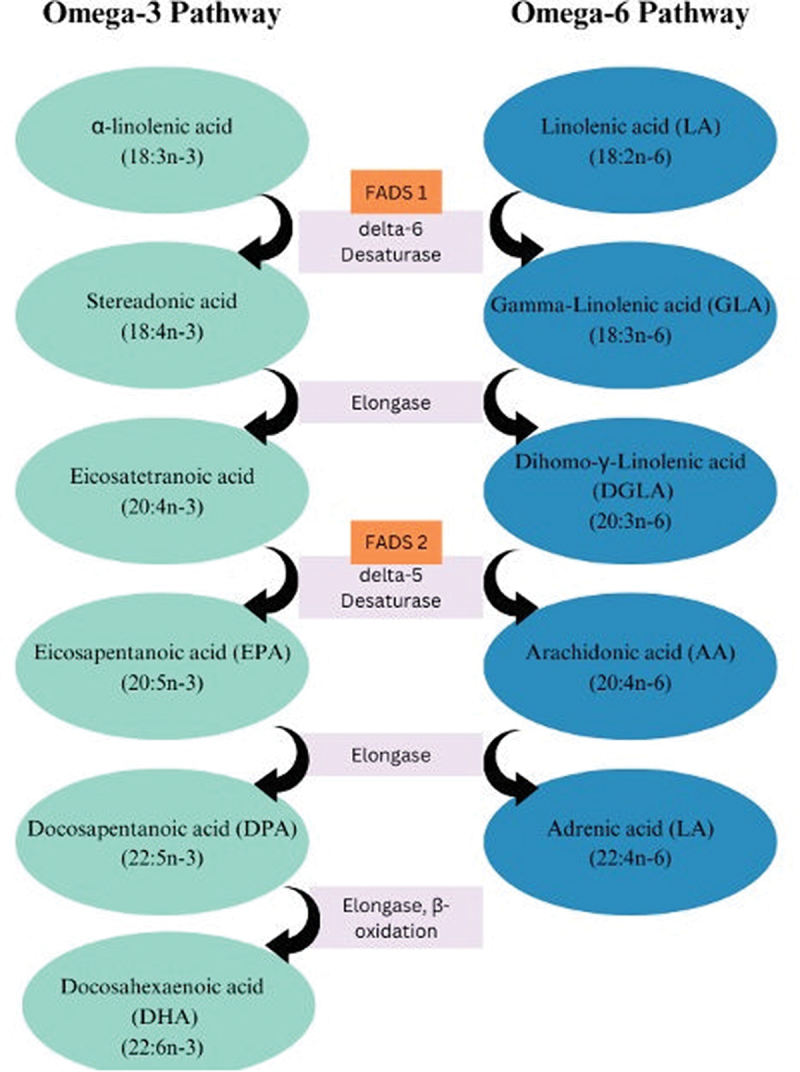
Omega-3 and omega-6 metabolic pathways.

Genetic polymorphisms involved in the synthesis of omega-3 PUFAs have been also found to impact blood levels of these FAs, resulting in differences in incorporation rates and effectiveness. Specifically, single nucleotide polymorphisms (SNPs) in genes encoding desaturase enzymes FADS1, encoding Δ-5 desaturase, and FADS2, encoding Δ-6 desaturase (Figure 2), are associated with lower EPA and DPA and higher ALA levels, and these SNPs could determine different conversion rates [44]. In addition, SNPs in *Elovl2* (ELOVL Fatty Acid Elongase 2) have been linked to higher EPA and DPA and lower DHA levels [45]. Carriers of the APOE + 4 polymorphism display decreased impact on their blood FA levels following EPA and DHA intake, indicating a dysfunctional DHA metabolism and fewer benefits from fish consumption [30,43,45]. Conversely, ALA supplementation elevates EPA and DPA levels but exerts a limited effect on plasmatic DHA levels and blood cell levels. On the contrary, supplementing with EPA results in increasing EPA and DPA levels by approximately 15 times more than ALA, yet it does not have a substantial impact on DHA levels. On the other hand, pre-formed DHA has been found to effectively increase DHA raise levels [46].

The impact of genetic polymorphisms mediating omega-3 biosynthesis has a significant impact on inflammatory processes and cognitive function. Studies on animal models have shown the benefits of omega-3 PUFAs intake in regulating inflammatory processes [47]. Omega-3 PUFAs modulate inflammation by modifying the cellular composition and receptors such as GPR120 (cell surface) and NR1C3 (PPAR-γ, intracellular) that regulate gene expression patterns and the signaling of inflammatory cells [48,49]. Deficiency in the production of anti-inflammatory mediators such as IL-10 could play a crucial role in cognitive decline in AD [47,50–52]. Thanks to the brain’s unique FA composition, high in palmitate, AA, and DHA, but low in EPA, genetic polymorphisms can determine the endogenous synthesis of omega-3. Supplementation with specific forms of omega-3 can improve these levels [27].

Because of the complexity of their integration and conversion in the human body, the metabolism of omega-3 PUFAs constitutes a challenge for clinical trials. The complex metabolism of omega-3 PUFAs is influenced by several variables, including differing absorption rates into various blood components and tissues, genetic variations in desaturase genes, and the unique fatty acid makeup of the brain. Future clinical trials must consider these difficulties and adopt a focused strategy for supplementation, concentrating on the EPA, DPA, or DHA forms to consistently raise blood levels of omega-3 PUFAs.

### 2.1. Sex differences in omega-3 synthesis and metabolism

Studies investigating sex differences in omega-3 metabolism have found around 15% higher DHA levels in women independent of dietary intake [53]. Mechanisms that may explain this include differences in beta-oxidation, body composition, and possible effects of sex hormones on the desaturation and elongation involved in omega-3 synthesis [28].

Studies using ALA breath samples have estimated that the 24-hour beta-oxidation rate of ALA is 33% in men and 22% in women. This difference may be due to women having lower muscle mass, which results in more ALA being available before it is used as a substrate for omega-3 synthesis [54,55]. Studies suggest that women tend to have higher levels of DPA and DHA in their adipose tissue, and during fasting periods their plasma NEFA (non-esterified fatty acid) levels reflect FAs from adipose tissue, with an almost twice as high concentration compared to men [56]. Additionally, sex-related differences in adipose tissue affect the circulating omega-3 levels, and overall synthesis [55]. Previous studies have reported differences in the effects of omega-3 supplementation between sexes. For instance, supplementation with fish oil (3g for 18 weeks) was found to enhance muscular function in women but not in men after resistance exercise intervention [57]. These sex differences have been attributed to variations in the enrichment of long-chain *n*-3 PUFAs in cell membranes [53].

The synthesis of EPA and DHA may be influenced by hormones. For example, omega-3 concentrations in rat plasma are positively correlated with estrogen and progesterone levels and negatively with testosterone [58]. A study conducted among transgender individuals revealed that those undergoing male-to-female transition and receiving estrogen and cyproterone acetate experienced higher concentrations of DHA in plasma. On the other hand, individuals transitioning from female to male individuals and taking testosterone had lower levels of DHA suggesting that estrogen stimulates the synthesis of DHA from ALA, while testosterone inhibits it [53].

In addition to affecting beta-oxidation, sex hormones may also influence enzymatic activity and potentially modify the desaturation and elongation processes necessary for omega-3 synthesis [28]. Previous research has demonstrated that estradiol upregulates fatty acid elongases Elovl2 and Elovl5 estradiol [59]. A study involving SH-SY5Y neuroblastoma cells treated with ALA and 17-β-estradiol revealed significant increases in EPA and DPA, but no significant effect on DHA production [60]. Studies on rats have shown that both estradiol and testosterone can increase D9-desaturase while decreasing D5 and D6-desaturase activities [58]. Additionally, testosterone therapy leads to a decrease in DHA content in plasma and liver lipids [28].]In support of these previous studies, evidence has also shown that women taking oral contraceptives exhibit higher rates of DHA production [61]. The upregulation of the desaturase and elongase enzymes by estradiol may account for this effect [59]. Nonetheless, these studies were limited by small sample sizes and a rarity of information concerning estrogen levels [55,58].

Despite the decrease of estrogen associated with menopause, postmenopausal women still have higher levels of circulating estrogen than males, potentially impacting DHA levels in later life, the presence of estrogen albeit in reduced levels plays a role in the transport and metabolism of fatty acids [62,63] A crossover clinical trial in this population administered 2.8 g of DHA in two 28-day intervention periods and showed that hormone replacement therapy can decrease the retro conversion of DHA to EPA from 9% to 5.2%, women on oral hormone replacement therapy could have increased levels of mitochondrial and peroxisomal fatty acid β-oxidation, the same study found a reduction in serum triacylglycerol of 20% and increases HDL-cholesterol by 8% in postmenopausal women, irrespective of hormone replacement therapy (HRT) status after DHA supplementation [28].

Due to the high heterogeneity in interventions and outcomes measured, it is challenging to determine the direction or magnitude of the effects. This highlights the importance of sex subgroup analysis planning in PUFA supplementation studies [28]. Further research is expected to provide more insight into the role of sex-specific factors, such as sex hormones, on omega-3 synthesis and circulating levels. Sex differences in omega-3 concentrations and synthesis pose a challenge for clinical trials using omega-3 supplements. Therefore, it is crucial to take these differences into account when designing clinical trials and interpreting results.

## 3. Dietary heterogeneity and evolution pose challenges for omega-3 supplementation in cognitive decline and dementia trials diet

There is significant heterogeneity in human diets across different populations. Generally, these diets have shifted from a 1:1 ratio of omega-6 to omega-3 fatty acids to a ratio of 15:1–16.7:1 in modern diets, indicating a deficiency in omega-3 PUFA [64]. The balance of omega-6 and omega-3 PUFAs is crucial for homeostasis [64]. For instance, the intake of omega-3 in the United States is lower than the recommended [65], with an intake at about 0.1% of total energy when compared with the recommended 0.6–1.2% of energy [66]. Low blood levels of omega-3 have been reported in several European countries including Ireland, the UK, Italy, Greece, Serbia, and Turkey, as well as in South America. Conversely, the Scandinavian countries and Japan have the highest blood levels of omega-3 [67]. To achieve the recommended levels, it is necessary to consume omega-3-rich fish at least twice a week, however, most people fail to meet this threshold [68]. Furthermore, animal models have shown that diets high in simple sugars can interfere with PUFAs metabolism, while high-fiber diets may prevent fats from being absorbed by the gastrointestinal tract [69,70]. These dietary factors pose challenges for clinical trials aimed at supplementing omega-3 in cognitive decline and dementia. Moreover, the dietary variability across populations can significantly influence the absorption and metabolism of these supplements [71], thereby affecting the outcomes of such trials. Future studies should include detailed dietary assessments, personalized dietary plans, dietary education, regular monitoring of dietary adherence, consideration of omega-3 bioavailability, and the inclusion of biomarkers to measure actual omega-3 status.

## 4. Populational studies and association of fish intake and Alzheimer’s disease risk

Epidemiological studies conducted in the U.S.A., Netherlands, and France have reinforced the connection between low fish and DHA intake and the risk of AD. These studies have shown a decrease in the risk of AD with the consumption of higher amounts of fish or omega-3 PUFAs [6,72–74]. Furthermore, research has confirmed that a diet rich in omega-3 or consuming fish with high omega-3 content is associated with a decreased likelihood of developing AD [74]. An additional study in Japan involved 185 participants aged over 80 years old without dementia demonstrated that serum DHA levels decreased with age and linked improved cognitive function to higher plasma levels of EPA and DHA+EPA [75]. Low omega-3 measured in erythrocytes has also been related to a significantly higher risk of cognitive impairment in a study of 720 older adults from Germany [76].

Populational studies indicate that diets high in omega-3 PUFAs are associated with a lower risk of neurodegenerative diseases. However, these results pose several limitations for future trials investigating the efficacy of omega-3 supplementation, as they often involve confounding factors such as diet, lifestyle, and genetic predispositions, which hinder the isolation of the effects of PUFAs supplementation. Furthermore, these studies frequently depend on self-reported dietary intake which may be susceptible to recall bias and thus produce inaccurate results. In addition, differing methods have been used to categorize fish and other food sources, thus rendering the complex and detailed interpretation of outcomes. To address these problems, forthcoming clinical trials need to employ accurate measurements of PUFAs intake. Future clinical trials will need to consider these specificities when designing study protocols and interpreting results.

## 5. The role of omega-3 FA in randomized trials

Although populational studies indicate that omega-3 intake has health-related benefits and reduces the risk of developing AD, inconsistent results from clinical trials (Table 1) raise concerns about the reproducibility of the effect found by observational studies.

The first randomized trial examining the impact of omega-3 FA on AD showed statistically significant differences in the Mini-Mental State Examination (MMSE) in the subgroup with very mild AD during the first 6 months (placebo group −2.6 points and intervention group −0.5 points; *p*2 = 0.01) using 1720 mg DHA + 600 mg EPA [33]. Chiu et al. [34] conducted a study with 46 participants with mild to moderate AD or mild cognitive impairment (MCI) who were given 1080 mg EPA + 720 mg DHA for 24 weeks. The results showed a statistically significant improvement in the Clinicians Global Impression of Change (CIBIC plus) 0.35 (IC95% 0.61 a 0.09, *p* = 0.008) vs placebo. Additionally, the subgroup with MCI experienced a significant delay in Alzheimer’s Disease Assessment Scale – Cognitive section (ADAS-cog) decline compared to the placebo (3.23 3.82 vs. 0.37 1.4, *p* = 0.03). Shinto et al. [39] investigated 39 participants with probable AD. The 12-month dietary intervention, consisting of 675 mg DHA and 975 mg EPA, did not yield any changes in ADAS-cog and ADL, nor were there any significant differences in MMSE scores. However, there was a significant difference observed in the group treated with omega-3 fatty acids in combination with lipoic acid, indicating that the dual therapy approach yielded better outcomes.

Further clinical trials have shown no statistically significant differences in the assessed outcomes. For instance, Quinn et al. [35] investigated 402 subjects taking DHA 2000 mg daily with mild to moderate AD and showed no differences in the rate of cognitive decline or cerebral volumes after 18 months of the study when compared to the placebo. In contrast, Scheltens et al. [36] found an increase in the Neuropsychological Test Battery (NTB) memory domain at 24 weeks for the intervention group supplemented with DHA 200 mg + EPA 300 mg, using the same compound Soininen et al. after 36 months reported significant reductions in decline were observed for several measures, including the NTB 5-item composite, Clinical Dementia Rating-Sum of Boxes, memory, and brain atrophy [37] On the other hand, Shah *et al..* [38] using the same product did not observe a significant reduction in cognitive decline as measured by ADAS-cog. In addition, Phillips et al. [40] supplementing with DHA 625 mg and EPA 600 mg EPA in 19 participants with AD found no significant results in MMSE after four months. Quinn et al. [35] aimed for a broader MMSE range (14–26), covering moderate to severe dementia. In contrast, Freud-Levi et al. focused on patients with MMSE scores exceeding 27, possibly targeting those with milder dementia [33]. Their results suggest that the effect of omega-3 May depend on the degree of cognitive decline.

Recently, Lin et al. [29] conducted a study to investigate the effects of omega-3 fatty acids on 90 elderly individuals. They found that providing a daily supplementation of 700 mg of DHA and 1600 mg of EPA daily or 350 mg of DHA and 800 mg of EPA for 24 months significantly improved ADAS-cog elements such as spoken language and constructional praxis. Additionally, improvements in circulatory chemokine CCL4 were also observed. In a similar work, Arellanes et al. [30] explored the effects of DHA and EPA supplementation on individuals at a high risk of AD. The cohort consisted of 152 adults who were given either a placebo or a daily dose of 2g of DHA and 400 mg of EPA daily. Although no differences in cognitive scores were reported after six months, the group receiving the DHA treatment exhibited an increase in DHA and EPA levels in their cerebrospinal fluid (CSF) compared to the placebo group. In a study by Torres-Mendoza et al. [31], participants were given either fish oil (450 mg of EPA and 1000 mg of DHA) or a placebo for a year. They observed that those taking fish oil had a significant decrease in oxidative stress markers and an increase in catalase, an antioxidant enzyme, at both 6 and 12-month intervals. These results were not observed in the placebo group, suggesting that omega-3 fatty acids may have antioxidant properties. In addition, produces oxylipins, which are oxidized products of PUFAs, particularly those derived from DHA and EPA. These oxylipins may serve as sensitive markers of cerebrovascular disease risk [77]. Soluble epoxide hydrolase (sEH) degrades these oxylipins. sEH inhibitors can decrease dihydroxyeicosatrienoic acid (DHET) levels and efficiently maintain endogenous epoxyeicosatrienoic acids (EET) levels. This change in focus toward sEH inhibitors emphasizes a new therapeutic direction that highlights the significance of omega-3 epoxides over individual PUFAs. This has potential implications for CNS, cardiovascular, and metabolic disorders [78].

Tamtaji et al. [32] conducted a 12-week randomized, double-blind, placebo-controlled trial in 40 subjects with Parkinson’s Disease (PD). Participants were assigned to receive either 1000 mg/day of omega-3 fatty acids sourced from flaxseed oil along with 400 IU/day of vitamin E supplements, or a placebo. The study investigated gene expression relating to inflammation, insulin, and lipids in peripheral blood mononuclear cells (PBMC) of PD patients using the RT-PCR method. Expression of the tumor necrosis factor-alpha (TNF-α) gene in PBMC was markedly reduced in the omega-3 intervention group. Moreover, compared with the placebo group, the treatment led to a considerable increase in the expression of the peroxisome proliferator-activated receptor gamma (PPAR-γ) gene and a significant downregulation of the expression of the oxidized low-density lipoprotein receptor (LDLR) gene.

Systematic reviews have also shown the effects of omega-3 supplementation on the risk of developing AD [4,79,80]. According to a meta-analysis by Mazereeuw et al. [79], supplementation with omega-3, irrespective of the dose, is beneficial for cognitive function (immediate recall, attention and processing speed) in patients with cognitive decline but without dementia. While Wu *et al..* [80] found that a higher fish intake may be associated with a lower risk of AD, they did not find the same association with omega-3 supplementation [34]. Zhang *et al..* [4] reported that the consumption of DHA consumption was linked to a reduced risk of dementia, without any dose-response relationship. Conversely, Burckhardt et al. [81] found no strong evidence for the advantages of omega-3 supplementation in AD [81].

As clinical trials have explored the impact of omega-3 supplementation on cognitive function, these have also explored their safety profiles, highlighting the safety of these supplements with no known drug-drug interactions [82] A recent systematic review highlighted that omega-3 supplementation in older adults could induce moderate adverse effects, mainly gastrointestinal disturbances, but they were not clinically significant. In general, omega-3 supplementation is safe [83].

### 5.1. Limitations of randomized trials

Randomized clinical trials (RCTs) have several limitations, including small sample sizes, and high participant dropout rates that may arise from caregivers’ lack of adherence and adverse gastrointestinal effects. Besides, inconsistent results can be attributed to the use of non-inert placebos like olive oil, concurrent medications, and varying dosages and durations.

Currently, there is no consensus on the ideal omega-3 supplementation dosage. RCTs have employed different DHA or DHA/EPA dosages, ranging from 240mg to 2.3g daily, with variable supplementation durations of 90 days to 18 months [35,84,85]. It is important to consider the ratio of DHA/EPA supplementation, as this can complicate the comparison of results and their extrapolation to the wider population [84]. Additionally, it is worth noting that the shorter duration of RCTs in comparison to life-long exposures observed in epidemiological studies can further add complexity to this area [74].

The limitations identified in RCTs present challenges for future trials. To overcome these challenges, future RCTs should increase sample sizes to provide more robust conclusions, minimize drop-out rates, standardize methodologies, use inert placebos to avoid confounding effects on the study outcome, include different stages of neurodegenerative diseases, and longer intervention duration. Large-scale randomized trials with extended interventions lasting 5 months and higher doses are necessary to fully assess the potential benefits of omega-3 supplementation on the brain. Trials with less than 1g per day of omega-3 May have reduced brain effects, particularly in individuals carrying the APOE4 gene variant [86].

## 6. Conclusions and future recommendations

Higher consumption of fish and omega-3 intake is associated with a reduced risk of cognitive decline, with EPA and DHA being linked to healthy aging, cardiovascular well-being, and reduced AD risk. Although erythrocyte FA content correlates more with intake than plasma, both are deemed appropriate biomarkers [20]. Omega-3 supplements have potential as an intervention for individuals at risk of neurodegenerative diseases such as AD. Evidence has shown that there is an increase in pro-inflammatory mediators two or more years before the onset of dementia, which could impact the progression to AD [33,87]. Therefore, it has been proposed that omega-3 FA supplementation could be beneficial in the early stages of neurodegenerative diseases [33].

## 7. Expert opinion

Research from observational suggests that omega-3 fatty acids are a promising therapeutic approach for interventions in neurodegenerative diseases; however, as highlighted in this review, the evidence from clinical trials has not shown consistent results, and there are several challenges for RCTs.

Firstly, the typical modern diet exposes individuals to low levels of omega-3, which can be exacerbated by the high consumption of carbohydrates, lipids, and fibers. These dietary elements can impair the metabolism of omega-3 Additionally, it is important to recognize several populational-based factors that make it challenging to isolate the impacts of omega-3 supplementation, including lifestyle choices and genetic polymorphisms. Furthermore, research methods pose additional challenges. For example, observational studies rely on dietary questionnaires, which frequently overlook to consider age and sex as biological variables, leading to inaccurate results, especially concerning omega-3 absorption, distribution, and metabolism. Consequently, the desired effects of omega-3 May not be fully achieved. Moreover, recall bias poses a risk, which can further complicate the comprehension and comparison of results.

Randomized placebo-controlled trials are thus required to understand the potential impact of omega-3 supplementation in the treatment or even prevention of neurodegenerative diseases. However, any effect is likely to be slight and gradually developing; thus, trials should be of sufficient duration and sample size. It is vital to utilize cost-effective trial strategies, which involve incorporating highly sensitive clinical outcomes such as novel digital technologies for detecting cognition and other relevant outcome measures. Moreover, to ensure target involvement and relevant biological effects, it is necessary to implement biomarkers.

The metabolism of omega-3 PUFAs is a complex challenge. These PUFAs can follow complex metabolic pathways with varying rates of incorporation into tissues, and genetic polymorphisms can influence the results of supplementation. APOE4 testing is also critical and should be considered before initiating omega-3 interventions. In addition, sex-dependent differences should not be overlooked. Differences in their omega-3 metabolism and synthesis must be considered when designing and analyzing the results of supplementation trials. This factor is critical to consider.

The goal of these interventions is to improve overall brain health, slow down cognitive decline, and improve the quality of life for those affected by the disease. Additionally, these interventions also aim to reduce inflammatory processes and oxidative stress which are crucial in the pathogenesis of diseases such as AD. Omega-3 PUFAs have demonstrated anti-inflammatory effects, although it may rely on the amount that reaches the brain and when it is administered. Given that there is no current treatment for most types of dementia, interventions that can delay the progression of the disease and improve the quality of life for those impacted are of paramount importance. Hence, omega-3 interventions offer a safe, affordable, noninvasive strategy to accomplish this objective.

The field of omega-3 supplementation research is expected to further advance its methods in the future, particularly regarding neurodegenerative diseases such as AD. Personalized medicine is set to become an essential approach, accounting for individual genetic polymorphisms, sex, age, dietary preferences, and lifestyle, and will permit tailoring of interventions to each person’s unique endophenotype. This area of research will also need to explore the potential synergies of omega-3 supplementation alongside other therapeutic interventions. Moreover, it is crucial to examine the formulation and distribution of omega-3 supplements to ensure their maximum absorption and bioavailability. Currently, the crossroads of genomics and omega-3 supplementation is fascinating. Understanding how genetic variants, such as the APOE4 allele, affect individual responses to omega-3 supplementation can help us design more effective and even more personalized interventions.

Finally, it is important to emphasize the need for a more personalized and targeted approach. For instance, prioritizing research into omega-3 formulations can increase levels of this PUFA not only in the blood but also in other organs, including the brain.

## 8. Recommendations for more personalized RCTs

- Daily dietary intake is a critical factor that must be considered when interpreting the results. A detailed analysis of the daily consumption of dietary nutrients by participants in trials, which may include the use of tools such as food frequency questionnaires (FFQs) correlated to plasma and erythrocyte biomarkers [20] helps to establish baseline levels of fatty acid intake. This preliminary information can be used to tailor more precise interventions and determine whether the observed effects are due to omega-3 supplementation.
- It is recommended to administer higher doses of the supplement over a longer period. The appropriate dosage will depend on the type of omega-3 supplement and its different formulations and may be related to a dose-response effect. Future RCTs should evaluate the effects of omega-3 at doses of at least 1g per day and extend the duration of the trials to 5 months or more.
- When designing future trials, it is essential to consider the inherent biological differences of each participant [28,44], including age, biological sex, and genetic polymorphisms such as *FADS1* and *FADS2*, which can influence the effectiveness of omega-3 supplements. By incorporating these variables into trials, we can evaluate the results more precisely and accurately, leading to a better understanding of the compound’s mechanism of action. Trials should consider the immunological profile of participants, including the evaluation of baseline inflammatory markers and assessment of immune system functionality.
- Strategies using omega-3 supplementation should also target the early stages of neurodegenerative diseases to produce impactful results. Omega-3 supplementation holds greater potential in the early stages of the disease, therefore RCTs should prioritize this population. The potential for the prevention of neurodegenerative diseases needs to be explored further.
- It is important to standardize the forms of omega-3 supplementation. Using identical formulations and sources of omega-3 supplements in all RCTs will facilitate a clearer comparison and more accurate interpretation of results.

## Article highlights

- Populational studies have linked high omega-3 diets with reduced risk of neurodegenerative diseases.
- The metabolism of omega-3 PUFAs can present challenges for supplementation trials, and a focused approach on EPA, DPA, or DHA is required to consistently elevate blood levels.
- Sex-specific factors such as hormones, immune system, and genetic polymorphisms can impact omega-3 metabolism, highlighting the need for more individualized assessments in PUFAs supplementation studies.
- Omega-3 supplementation can modulate the immune response in neurodegenerative diseases, promoting anti-inflammatory states, enhancing phagocytic activity, and reducing inflammasome activation.
- Supplementation trials face challenges due to dietary heterogeneity and variations in the omega-6 to omega-3 ratio.
- The limitations of randomized clinical trials, including small sample sizes, high dropout rates, and varied interventions, underscore the necessity for meticulous design and accounting for confounding variables to ensure consistent outcomes in omega-3 supplementation research.
